# Supplementary figures and images for: Concurrent anemia and stunting among schoolchildren in Wonago district in southern Ethiopia: a cross-sectional multilevel analysis
Source: PeerJ. 2021 May 6;9:e11158. doi: 10.7717/peerj.11158 (PMC8106909; doi:10.7717/peerj.11158)

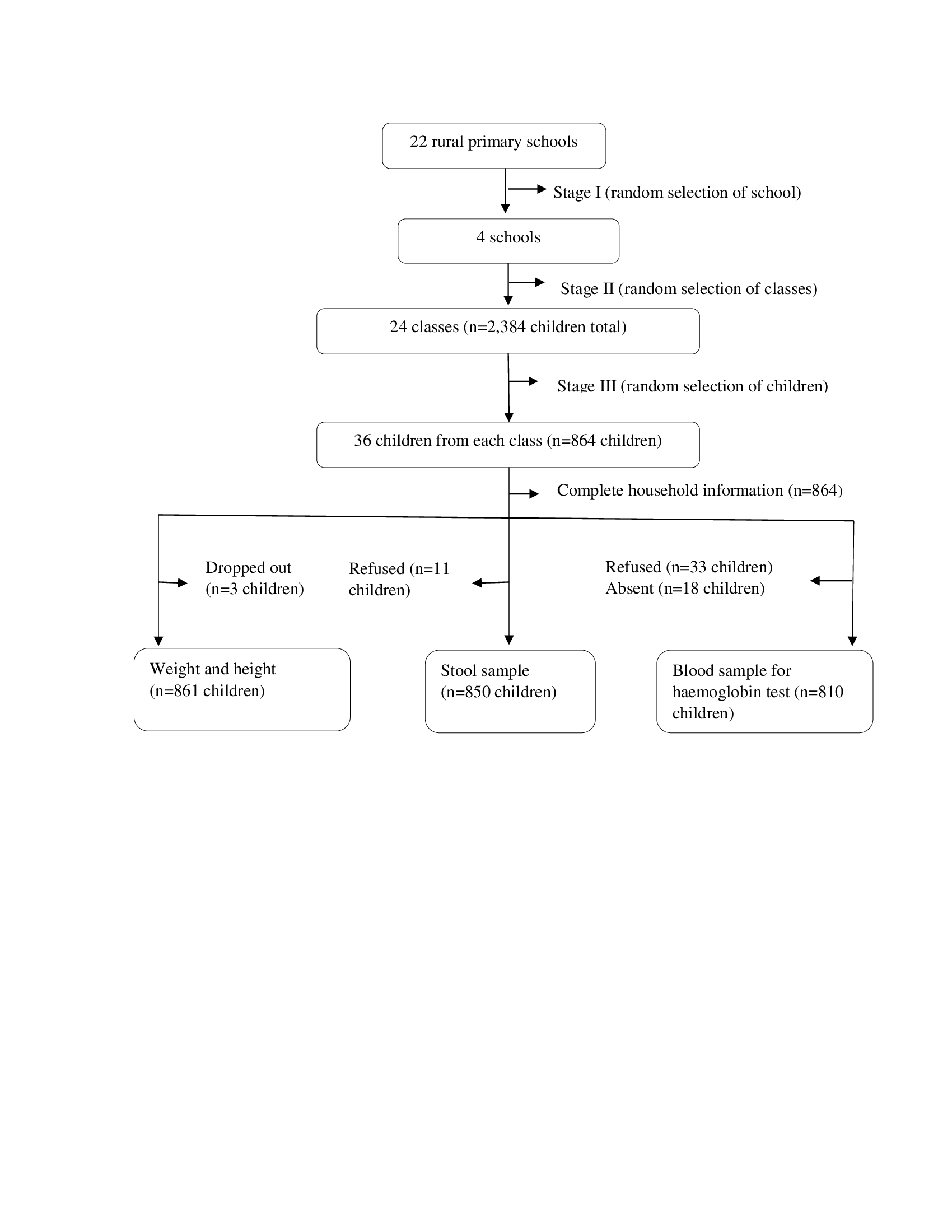

Supplement: Supplemental Information 1 [file peerj-09-11158-s001.png]
