# Supplementary material for: Concurrent anemia and stunting among schoolchildren in Wonago district in southern Ethiopia: a cross-sectional multilevel analysis
Source: PeerJ. 2021 May 6;9:e11158. doi: 10.7717/peerj.11158 (PMC8106909; doi:10.7717/peerj.11158)
Supplement: Supplemental Information 3 [file peerj-09-11158-s003.docx]

**Table S2** **Food groups and beverages consumed by schoolchildren the day before the survey in the Wonago district of southern Ethiopia, 2017 (n=861)**

| **Food group** | | **Frequency** | **Percent** |
| --- | --- | --- | --- |
| Group 1. | Cereals, root or tubers (Maize, wheat, teff, rice, white potato, kocho or bulla*) | 861 | 100 |
| Group 2. | Vitamin A rich plant foods (Carrot, sweat potato, papaya, mango) | 85 | 9.9 |
| Group 3. | Other fruits (avocado, orange) | 160 | 18.6 |
| Group 4. | Other vegetables (tomato, cabbage, onion) | 195 | 22.6 |
| Group 5. | Meat or fish | 35 | 4.1 |
| Group 6. | Eggs | 147 | 17.1 |
| Group 7. | Pulses or legumes (beans, peas, lentils) | 404 | 46.9 |
| Group 8. | Milk and milk products (milk, yogurt) | 129 | 15.0 |
| Group 9. | Food cooked in oils or fats | 32 | 3.7 |
| Coffee or tea |  | 749 | 87.0 |

* Kocho or bulla: Flour made of the root and bark of *ensete or wasa.*
