# Supplementary material for: Concurrent anemia and stunting among schoolchildren in Wonago district in southern Ethiopia: a cross-sectional multilevel analysis
Source: PeerJ. 2021 May 6;9:e11158. doi: 10.7717/peerj.11158 (PMC8106909; doi:10.7717/peerj.11158)
Supplement: Supplemental Information 5 [file peerj-09-11158-s005.docx]

**Table S4 Prevalence of anemia, stunting, thinness, and underweight, among schoolchildren in the Wonago district, southern Ethiopia, 2017**

| **Variables** | **Frequency** | **Percent** |
| --- | --- | --- |
| Non anemic | 570 | 70.4 |
| Anemic | 240 | 29.6 |
| Mild anemia | 204 | 85 |
| Moderate anemia | 36 | 15 |
| Not stunted | 583 | 67.7 |
| Stunted | 278 | 32.3 |
| Severly stunted | 104 | 37.4 |
| Not thin | 776 | 90.1 |
| Thin | 85 | 9.9 |
| Not underweight | 247 | 81.3 |
| Underweight | 57 | 18.7 |
| Severly underweight | 3.3 | 10 |
