# Supplementary material for: Concurrent anemia and stunting among schoolchildren in Wonago district in southern Ethiopia: a cross-sectional multilevel analysis
Source: PeerJ. 2021 May 6;9:e11158. doi: 10.7717/peerj.11158 (PMC8106909; doi:10.7717/peerj.11158)
Supplement: Supplemental Information 6 [file peerj-09-11158-s006.docx]

**Table S5 The proportion of anemia, stunting, and CAS in relation to individual, household, and school factors among schoolchildren in the Wonago district of southern Ethiopia, 2017**

| **Variables** | |  | **Anemia** | |  | **Stunting** | |  | **CAS** | |
| --- | --- | --- | --- | --- | --- | --- | --- | --- | --- | --- |
| **Individual child factors** | | **N** | **Yes (%)** | **No (%)** | **N** | **Yes (%)** | **No (%)** | **N** | **Yes (%)** | **No (%)** |
| Sex | Boys | 456 | 134 (29.4) | 322 (70.6) | 483 | 167 (34.6) | 316 (65.4) | 456 | 44 (9.6) | 412 (90.4) |
|  | Girls | 354 | 106 (29.9) | 248 (70.1) | 378 | 111 (29.4) | 267 (70.6) | 354 | 41 (11.6) | 313 (88.4) |
| Age in years | 7-9 | 151 | 61 (40.4) | 90 (59.6) | 158 | 25 (15.8) | 133 (84.2 | 151 | 11 (7.3) | 140 (92.7) |
|  | 10-14 | 659 | 179 (27.2) | 480 (72.8) | 703 | 253 (36.0) | 450 (64.0) | 659 | 74 (11.2) | 585 (88.8) |
| Trim nails every week | Yes | 623 | 191 (30.7) | 432 (69.3) | 665 | 200 (30.1) | 465 (69.9) | 623 | 65 (10.4) | 558 (89.6) |
|  | No | 187 | 49 (26.2) | 138 (73.8) | 196 | 78 (39.8) | 118 (60.2) | 187 | 20 (10.7) | 167 (89.3) |
| Hand-washing with soap after use of latrine | Always | 97 | 21 (21.6) | 76 (78.4) | 103 | 22 (21.4) | 81 (78.6) | 97 | 6 (6.2) | 91 (93.8) |
|  | Sometimes or not always | 467 | 111 (23.8) | 356 (76.2) | 487 | 167 (34.3) | 320 (65.7) | 467 | 45 (9.6) | 422 (90.4) |
|  | Never | 246 | 108 (43.9) | 138 (56.1) | 271 | 89 (32.8) | 182 (67.2) | 246 | 34 (13.8) | 212 (86.2) |
| Walking barefoot | Always | 20 | 8 (40.0) | 12 (60.0) | 22 | 10 (45.4) | 12 (54.6) | 20 | 6 (30.0) | 14 (70.0) |
|  | Sometimes | 381 | 112 (29.4) | 269 (70.6) | 400 | 139 (34.7) | 261 (65.3) | 381 | 40 (10.5) | 341 (89.5) |
|  | Never | 409 | 120 (29.3) | 289 (70.7) | 439 | 129 (29.4) | 310 (70.6) | 409 | 39 (9.5) | 370 (90.5) |
| Taking regular meals before attending school | Yes | 752 | 219 (29.1) | 533 (70.9) | 798 | 261 (32.7) | 537 (67.3) | 752 | 80 (10.6) | 672 (89.4) |
|  | No | 58 | 21 (36.2) | 37 (63.8) | 63 | 17 (27.0) | 46 (73.0) | 58 | 5 (8.6) | 53 (91.4) |
| Reported illness in the past one month | Yes | 35 | 12 (34.3) | 23 (65.7) | 37 | 10 (27.0) | 27 (73.0) | 35 | 2 (5.7) | 33 (94.3) |
|  | No | 775 | 228 (29.4) | 547 (70.6) | 824 | 268 (32.5) | 556 (67.5) | 775 | 228 (29.4) | 692 (89.3) |
| Anemia | No | - | - | - | 570 | 170 (29.8) | 400 (70.2) | - | - | - |
|  | Yes | - | - | - | 240 | 85 (35.4) | 155 (64.6) | - | - | - |
| Stunting | No | 555 | 155 (27.9) | 400 (72.1) | - | - | - | - | - | - |
|  | Yes | 255 | 85 (33.3) | 170 (66.7) | - | - | - | - | - | - |
| *A. lumbricoides* | No | 649 | 174 (26.8) | 475 (73.2) | 691 | 223 (32.3) | 468 ()67.7 | 649 | 60 (9.2) | 589 (90.8) |
|  | Yes | 156 | 64 (41.0) | 92 (59.0) | 159 | 49 (30.8) | 110 (69.2) | 156 | 23 (14.7) | 133 (85.3) |
| *T. trichiura* | No | 465 | 123 (26.5) | 342 (73.5) | 490 | 153 (31.2) | 337 (68.8) | 465 | 41 (8.8) | 424 (91.2) |
|  | Yes | 340 | 115 (33.8) | 225 (66.2) | 360 | 119 (33.1) | 241 (66.9) | 340 | 42 (12.4) | 298 (87.6) |
| Hookworm | No | 268 | 221 (28.8) | 547 (71.2) | 813 | 260 (32.0) | 553 (68.0) | 768 | 78 (10.2) | 690 (89.8) |
|  | Yes | 37 | 17 (45.9) | 20 (54.1) | 37 | 12 (32.4) | 25 (67.6) | 37 | 5 (13.5) | 32 (86.5) |

CAS; concurrent anemia and stunting; N: total number of children

**Table S5 The proportion of anemia, stunting, and CAS in relation to individual, household, and school factors among schoolchildren in the Wonago district of Southern Ethiopia, 2017 (Continued)**

| **Variables** | |  | **Anemia** | |  | **Stunting** | |  | **CAS** | |
| --- | --- | --- | --- | --- | --- | --- | --- | --- | --- | --- |
|  |  | **N** | **Yes (%)** | **No (%)** | **N** | **Yes (%)** | **No (%)** | **N** | **Yes (%)** | **No (%)** |
| Received de-worming treatment in the past 6 months | Yes | 174 | 66 (37.9) | 108 (62.1) | 191 | 59 (30.9) | 132 (69.1) | 174 | 21 (12.1) | 153 (87.9) |
|  | No | 636 | 174 (27.4) | 462 (72.6) | 670 | 219 (32.7) | 451 (67.3) | 636 | 64 (10.1) | 572 (89.9) |
| Head lice | Yes | 315 | 101 (32.1) | 214 (67.9) | 342 | 123 (36.0) | 219 (64.0) | 315 | 42 (13.3) | 273 (86.7) |
|  | No | 495 | 139 (28.1) | 356 (71.9) | 519 | 155 (29.9) | 364 (70.1) | 495 | 43 (8.7) | 452 (91.3) |
| **Individual parent factors** | |  |  |  |  |  |  |  |  |  |
| Mother’s education | No formal education | 718 | 216 (30.1) | 502 (69.9) | 761 | 250 (32.8) | 511 (67.2) | 718 | 79 (11.0) | 639 (89.0) |
|  | Primary and above | 88 | 24 (27.3) | 64 (72.7) | 96 | 27 (28.1) | 69 (71.9) | 88 | 6 (6.8) | 82 (93.2) |
| Father’s education | No formal education | 391 | 122 (31.2) | 269 (68.8) | 420 | 140 (33.3) | 280 (66.7) | 391 | 46 (11.8) | 345 (88.2) |
|  | Primary and above | 359 | 103 (28.7) | 256 (71.3) | 380 | 119 (31.3) | 261 (68.7) | 359 | 33 (9.2) | 326 (90.8) |
| **Household factors** | |  |  |  |  |  |  |  |  |  |
| Wealth | Poor | 273 | 73 (26.7) | 200 (73.3) | 287 | 104 (36.2) | 183 (63.8) | 273 | 30 (11) | 243 (89.0) |
|  | Middle | 275 | 92 (33.4) | 183 (66.6) | 297 | 88 (29.6) | 209 (70.4) | 275 | 28 (10.2) | 247 (89.8) |
|  | Rich | 262 | 75 (28.6) | 187 (71.4) | 277 | 86 (31.1) | 191 (68.9) | 262 | 27 (10.3) | 235 (89.7) |
| Family size | 1-4 | 76 | 21 (27.6) | 55 (72.4) | 78 | 19 (24.4) | 59 (75.6) | 76 | 4 (5.3) | 72 (94.7) |
|  | ≥5 | 734 | 219 (29.8) | 515 (70.2) | 783 | 259 (33.1) | 524 (66.9) | 734 | 81 (11.0) | 653 (89.0) |
| Using treated water at home | Yes | 98 | 32 (32.6) | 66 (67.4) | 109 | 22 (20.2) | 87 (79.8) | 98 | 4 (4.1) | 94 (95.9) |
|  | No | 712 | 208 (29.2) | 504 (70.8) | 752 | 256 (34.0) | 496 (66.0) | 712 | 81 (11.4) | 631 (88.6) |
| Food insecurity | No | 401 | 110 (27.4) | 291 (72.6) | 424 | 149 (35.1) | 275 (64.9) | 401 | 43 (10.7) | 358 (89.3) |
|  | Yes | 409 | 130 (31.8) | 279 (68.2) | 437 | 129 (29.5) | 308 (70.5) | 409 | 42 (10.3) | 367 (89.7) |
| Received food aid in the past 6 months | No | 767 | 227 (29.6) | 540 (70.4) | 814 | 270 (33.2) | 544 (66.8) | 767 | 83 (10.8) | 684 (89.2) |
|  | Yes | 43 | 13 (30.2) | 30 (69.8) | 47 | 8 (17.0) | 39 (83.0) | 43 | 2 (4.6) | 41 (95.4) |
| **School factors** | |  |  |  |  |  |  |  |  |  |
| Participates in school feeding program | No | 400 | 152 (38.0) | 248 (62.0) | 431 | 139 (32.3) | 292 (67.7) | 400 | 55 (13.7) | 345 (86.3) |
|  | Yes | 410 | 88 (21.5) | 322 (78.5) | 430 | 139 (32.3) | 291 (67.7) | 410 | 30 (7.3) | 380 (92.7) |

CAS; concurrent anemia and stunting; N: total number of children
