# Supplementary material for: Concurrent anemia and stunting among schoolchildren in Wonago district in southern Ethiopia: a cross-sectional multilevel analysis
Source: PeerJ. 2021 May 6;9:e11158. doi: 10.7717/peerj.11158 (PMC8106909; doi:10.7717/peerj.11158)
Supplement: Supplemental Information 7 [file peerj-09-11158-s007.docx]

**Table S6 Bivariate, multilevel, mixed-effect, regression analysis of anemia, stunting, and CAS among schoolchildren in the Wonago district of southern Ethiopia, 2017**

| **Variables** | | **Crude odds ratio (COR) (95% CI)** | | | | | |
| --- | --- | --- | --- | --- | --- | --- | --- |
| **Individual child factors** | | **Anemia** | **P-value** | **Stunting** | **P-value** | **CAS** | **P-value** |
| Sex | Boys | 1.01 (0.73, 1.39) | 0.954 | 1.28 (0.96, 1.72) | 0.094 | 0.81 (0.5, 1.29) | 0.376 |
|  | Girls | 1.0 |  | 1.0 |  | 1.0 |  |
| Age in years | Mean (SD) | 0.94 (0.84, 1.04) | 0.212 | 1.41 (1.21, 1.65) | 0.000 | 1.36 (1.13, 1.65) | 0.001 |
| Nail trimming every week | Yes | 0.79 (0.46, 1.37) | 0.407 | 0.65 (0.47, 0.91) | 0.011 | 0.61 (0.28, 1.32) | 0.211 |
|  | No | 1.0 |  | 1.0 |  | 1.0 |  |
| Hand-washing with soap after latrine | Always | 1.0 |  | 1.0 |  | 1.0 |  |
|  | Sometimes or not always | 1.40 (0.78, 2.53) | 0.258 | 1.92 (1.16, 3.19) | 0.012 | 1.74 (0.68, 4.44) | 0.249 |
|  | Never | 2.17 (1.13, 4.20) | 0.020 | 1.79 (1.05, 3.08) | 0.032 | 1.67 (0.58, 4.81) | 0.342 |
| Walking bare foot | Always | 1.69 (0.62, 4.59) | 0.303 | 2.01 (0.84, 4.79) | 0.115 | 5.36 (1.74, 16.5) | 0.003 |
|  | Sometimes | 0.99 (0.70, 1.41) | 0.980 | 1.28 (0.95, 1.72) | 0.099 | 1.16 (0.70, 1.91) | 0.573 |
|  | Never | 1.0 |  | 1.0 |  | 1.0 |  |
| Taking a meal regularly before attending school | Yes | 0.51 (0.28, 0.94) | 0.030 | 1.31 (0.73, 2.33) | 0.363 | 0.94 (0.34, 2.56) | 0.901 |
|  | No | 1.0 |  | 1.0 |  | 1.0 |  |
| Reported illness in the past one month | Yes | 1.35 (0.64, 2.87) | 0.432 | 0.77 (0.37, 1.61) | 0.487 | 0.51 (0.12, 2.23) | 0.372 |
|  | No | 1.0 |  | 1.0 |  | 1.0 |  |
| Anemia | No | - |  | 1.0 |  | - |  |
|  | Yes | - |  | 1.32 (0.95, 1.84) | 0.102 | - |  |
| Stunting | No | 1.0 |  | - |  | - |  |
|  | Yes | 1.36 (0.97, 1.93) | 0.077 | - |  | - |  |
| Thinness | No | 1.0 |  | - |  | - |  |
|  | Yes | 1.04 (0.61, 1.78) | 0.870 | - |  | - |  |
| *A. lumbricoides* | No | 1.0 |  | 1.0 |  | 1.0 |  |
|  | Yes | 1.81 (1.22, 2.68) | 0.003 | 0.94 (0.64, 1.36) | 0.724 | 1.66 (0.97, 2.86) | 0.064 |
| T. trichiura | No | 1.0 |  | 1.0 |  | 1.0 |  |
|  | Yes | 1.56 (1.13, 2.17) | 0.008 | 1.09 (0.82, 1.47) | 0.545 | 1.59 (0.99, 2.55) | 0.053 |
| Hookworm | No | 1.0 |  | 1.0 |  | 1.0 |  |
|  | Yes | 2.02 (0.98, 4.12) | 0.055 | 1.02 (0.50, 2.07) | 0.951 | 1.32 (0.48, 3.59) | 0.591 |
| DDS |  | 0.99 (0.87, 1.12) | 0.889 | 0.97 (0.87, 1.07) | 0.564 | 0.92 (0.77, 1.11) | 0.394 |
| Received de-worming treatment in the past 6 months | Yes | 1.42 (0.87, 2.32) | 0.160 | 0.93 (0.64, 1.34) | 0.693 | 1.33 (0.65, 2.72) | 0.432 |
|  | No | 1.0 |  | 1.0 |  | 1.0 |  |
| Head lice | Yes | 1.22 (0.87, 1.70) | 0.254 | 1.33 (0.99, 1.78) | 0.058 | 1.69 (1.05, 2.74) | 0.031 |
|  | No | 1.0 |  | 1.0 |  | 1.0 |  |

CAS**:** Concurrent of anemia and stunting; CI: confidence interval; DDS: Dietary Diversity score; OR: odds ratio

**Table S6 Bivariate, multilevel, mixed-effect, regression analysis of anemia, stunting, and CAS among schoolchildren in the Wonago district of southern Ethiopia, 2017 (Continued)**

| **Variables** | | **Crude odds ratio (COR) (95% CI)** | | | | | |
| --- | --- | --- | --- | --- | --- | --- | --- |
| **Individual parent factors** | | **Anemia** | **P-value** | **Stunting** | **P-value** | **CAS** | **P-value** |
| Mother’s education | No formal education | 1.39 (0.81, 2.43) | 0.234 | 1.35 (0.82, 2.22) | 0.243 | 2.33 (0.93, 5.84) | 0.071 |
|  | Primary and above | 1.0 |  | 1.0 |  | 1.0 |  |
| Father’s education | No formal education | 0.95 (0.66, 1.36) | 0.768 | 1.12 (0.82, 1.52) | 0.482 | 1.31 (0.78, 2.19) | 0.309 |
|  | Primary and above | 1.0 |  | 1.0 |  | 1.0 |  |
| **Household factors** | |  |  |  |  |  |  |
| Wealth | Poor | 0.86 (0.57, 1.28) | 0.450 | 1.27 (0.89, 1.81) | 0.185 | 1.03 (0.58, 1.83) | 0.917 |
|  | Middle | 1.03 (0.68, 1.55) | 0.890 | 0.94 (0.66, 1.35) | 0.746 | 0.80 (0.44, 1.47) | 0.472 |
|  | Rich | 1.0 |  | 1.0 |  | 1.0 |  |
| Family size | 1-4 | 1.0 |  | 1.0 |  | 1.0 |  |
|  | ≥5 | 1.09 (0.62, 1.91) | 0.758 | 1.53 (0.89, 2.63) | 0.121 | 2.08 (0.73, 5.94) | 0.172 |
| Using treated drinking water | Yes | 0.97 (0.59, 1.59) | 0.920 | 0.49 (0.29, 0.79) | 0.004 | 0.28 (0.10, 0.80) | 0.017 |
|  | No | 1.0 |  | 1.0 |  | 1.0 |  |
| Food insecurity | No | 1.0 |  | 1.0 |  | 1.0 |  |
|  | Yes | 1.25 (0.83, 1.88) | 0.278 | 0.77 (0.58, 1.03) | 0.078 | 1.001 (0.56, 1.79) | 0.996 |
| Received food aid in the past 6 months | No | 1.0 |  | 1.0 |  | 1.0 |  |
|  | Yes | 0.71 (0.34, 1.48) | 0.362 | 0.42 (0.19, 0.90) | 0.027 | 0.33 (0.10, 1.45) | 0.142 |
| **School factors** | |  |  |  |  |  |  |
| Participates in school meal programme | No | 1.0 |  | 1.0 |  | 1.0 |  |
|  | Yes | 0.44 (0.18, 1.12) | 0.087 | 1.003 (0.74, 1.35) | 0.984 | 0.48 (0.22, 1.03) | 0.059 |

CAS**:** Concurrent of anemia and stunting; CI: confidence interval; DDS: Dietary Diversity score; OR: odds ratio
