# Supplementary material for: Concurrent anemia and stunting among schoolchildren in Wonago district in southern Ethiopia: a cross-sectional multilevel analysis
Source: PeerJ. 2021 May 6;9:e11158. doi: 10.7717/peerj.11158 (PMC8106909; doi:10.7717/peerj.11158)
Supplement: Supplemental Information 8 [file peerj-09-11158-s008.docx]

**Table S7 Multivariate, multilevel, mixed-effect, regression analysis of CAS among schoolchildren in the Wonago district of southern Ethiopia, 2017 (n=810)**

| **Variables** | | **CAS** | | **Adjusted OR (95% CI)** | | | | | | | | | | |
| --- | --- | --- | --- | --- | --- | --- | --- | --- | --- | --- | --- | --- | --- | --- |
|  |  |  |  | **Models** | | | | | | | | | | |
| **Individual child factors** | | **Yes (%)** | **No (%)** | **I** | **II** | **P-value** | **III** | **P-value** | **IV** | **P-value** | **V** | **P-value** | **VI** | **P-value** |
| Sex | Boys | 44 (9.6) | 412 (90.4) | - | 0.84 (0.51, 1.38) | 0.502 | - |  | - |  | - |  | 0.84 (0.51, 1.39) | 0.499 |
|  | Girls | 41 (11.6) | 313 (88.4) | - | 1.0 |  | - |  | - |  | - |  | 1.0 |  |
| Age in years | Mean (SD) | 11.4 (1.9) |  | - | 1.36 (1.11, 1.67) | 0.003 | - |  | - |  | - |  | 1.39 (1.13, 1.71) | 0.002 |
| Trim nail every week | Yes | 65 (10.4) | 558 (89.6) | - | 0.68 (0.30, 1.57) | 0.373 | - |  | - |  | - |  | - |  |
|  | No | 20 (10.7) | 167 (89.3) | - | 1.0 |  | - |  | - |  | - |  | - |  |
| Hand-washing with soap after use of latrine | Always | 6 (6.2) | 91 (93.8) | - | 1.0 |  | - |  | - |  | - |  | 1.0 |  |
|  | Sometimes or not always | 45 (9.6) | 422 (90.4) | - | 4.10 (1.17, 14.1) | 0.027 | - |  | - |  | - |  | 4.30 (1.21, 15.3) | 0.024 |
|  | Never | 34 (13.8) | 212 (86.2) | - | 3.35 (0.89, 12.6) | 0.074 | - |  | - |  | - |  | 3.10 (0.82, 11.8) | 0.097 |
| Walking bare foot | Always | 6 (30.0) | 14 (70.0) | - | 8.24 (2.24, 30.4) | 0.002 | - |  | - |  | - |  | 10.4 (2.77, 39.1) | 0.001 |
|  | Sometimes | 40 (10.5) | 341 (89.5) | - | 1.13 (0.66, 1.92) | 0.660 | - |  | - |  | - |  | 1.18 (0.68, 2.05) | 0.552 |
|  | Never | 39 (9.5) | 370 (90.5) | - | 1.0 |  | - |  | - |  | - |  | 1.0 |  |
| *A. lumbricoides* | No | 60 (9.2) | 589 (90.8) | - | 1.0 |  | - |  | - |  | - |  | - |  |
|  | Yes | 23 (14.7) | 133 (85.3) | - | 1.40 (0.78, 2.53) | 0.259 | - |  | - |  | - |  | - |  |
| *T. trichiura* | No | 41 (8.8) | 424 (91.2) | - | 1.0 |  | - |  | - |  | - |  | 1.0 |  |
|  | Yes | 42 (12.4) | 298 (87.6) | - | 1.70 (1.03, 2.82) | 0.038 | - |  | - |  | - |  | 1.74 (1.05, 2.88) | 0.030 |
| Head lice | Yes | 42 (13.3) | 273 (86.7) | - | 1.64 (0.98, 2.73) | 0.060 | - |  | - |  | - |  | 1.71 (1.007, 2.92) | 0.047 |
|  | No | 43 (8.7) | 452 (91.3) | - | 1.0 |  | - |  | - |  | - |  | 1.0 |  |
| **Individual parent factors** | |  |  |  |  |  |  |  |  |  |  |  |  |  |
| Mother’s education | No formal education | 79 (11.0) | 639 (89.0) | - | - |  | 2.33 (0.93, 5.84) | 0.071 | - |  | - |  | 2.43 (0.90, 6.57) | 0.081 |
|  | Primary and above | 6 (6.8) | 82 (93.2) | - | - |  | 1.0 |  | - |  | - |  | 1.0 |  |
| **Household factors** | |  |  |  |  |  |  |  |  |  |  |  |  |  |
| Wealth | Poor | 30 (11) | 243 (89.0) | - | - |  | - |  | 1.04 (0.58, 1.85) | 0.901 | - |  | 1.06 (0.56, 1.98) | 0.863 |
|  | Middle | 28 (10.2) | 247 (89.8) | - | - |  | - |  | 0.79 (0.42, 1.47) | 0.455 | - |  | 0.89 (0.45, 1.75) | 0.732 |
|  | Rich | 27 (10.3) | 235 (89.7) | - | - |  | - |  | 1.0 |  | - |  | 1.0 |  |
| Family size | 1-4 | 4 (5.3) | 72 (94.7) | - | - |  | - |  | 1.0 |  | - |  | 1.0 |  |
|  | ≥5 | 81 (11.0) | 653 (89.0) | - | - |  | - |  | 2.13 (0.74, 6.12) | 0.160 | - |  | 2.04 (0.68, 6.15) | 0.203 |
| Using treated water at home | Yes | 4 (4.1) | 94 (95.9) | - | - |  | - |  | 0.29 (0.10, 0.84) | 0.022 | - |  | 0.32 (0.11, 0.97) | 0.043 |
|  | No | 81 (11.4) | 631 (88.6) | - | - |  | - |  | 1.0 |  | - |  | 1.0 |  |
| Received food aid in the past 6 months | No | 83 (10.8) | 684 (89.2) | - | - |  | - |  | 1.0 |  | - |  | 1.0 |  |
|  | Yes | 2 (4.6) | 41 (95.4) | - | - |  | - |  | 0.34 (0.07, 1.53) | 0.158 | - |  | 0.26 (0.05, 1.28) | 0.097 |

CAS: concurrent anemia and stunting; CI: confidence interval; OR: odds ratio

**Table S7 Multivariate, multilevel, mixed-effect, regression analysis of CAS among schoolchildren in the Wonago district of southern Ethiopia, 2017 (Continued)**

| **Variables** | | **CAS** | | **Adjusted OR (95% CI)** | | | | | | | | | | |
| --- | --- | --- | --- | --- | --- | --- | --- | --- | --- | --- | --- | --- | --- | --- |
|  |  |  |  | **Models** | | | | | | | | | | |
| **School factors** | | **Yes (%)** | **No** | **I** | **II** | **P-value** | **III** | **P-value** | **IV** | **P-value** | **V** | **P-value** | **VI** | **P-value** |
| Participates in school feeding program | No | 55 (13.7) | 345 (86.3) |  | - |  | - |  | - |  | 1.0 |  | 1.0 |  |
|  | Yes | 30 (7.3) | 380 (92.7) |  | - |  | - |  | - |  | 0.48 (0.22, 1.03) | 0.059 | 0.29 (0.07, 1.17) | 0.082 |
| **Variation and model fitness** | |  |  |  |  |  |  |  |  |  |  |  |  |  |
| Variance | School level |  |  | 0.17 | 0.44 |  | 0.20 |  | 0.24 |  | 0.04 |  | 0.27 |  |
|  | Class level |  |  | 0.33 | 0.51 |  | 0.37 |  | 0.32 |  | 0.32 |  | 0.50 |  |
| Intra-cluster correlation | School |  |  | 4.5% | 10.4% |  | 5.2% |  | 6.45 |  | 1% |  | 6.8% |  |
|  | Class |  |  | 13.2% | 22.4% |  | 14.8% |  | 14.6% |  | 9.8% |  | 19% |  |
| **Model fitness** | |  |  |  |  |  |  |  |  |  |  |  |  |  |
| -2 Log likelihood | |  |  | 530 | 484 |  | 525 |  | 516 |  | 527 |  | 466 |  |
| Akaike information criterion | |  |  | 536 | 510 |  | 533 |  | 532 |  | 535 |  | 502 |  |
| Area under the curve=0.81 | |  |  |  |  |  |  |  |  |  |  |  |  |  |

CAS: concurrent anemia and stunting; CI: confidence interval; OR: odds ratio
