# Supplementary material for: Concurrent anemia and stunting among schoolchildren in Wonago district in southern Ethiopia: a cross-sectional multilevel analysis
Source: PeerJ. 2021 May 6;9:e11158. doi: 10.7717/peerj.11158 (PMC8106909; doi:10.7717/peerj.11158)
Supplement: Supplemental Information 9 [file peerj-09-11158-s009.docx]

**Table S8 Multivariate, multilevel, mixed-effect, regression analysis of anemia among schoolchildren in the Wonago district of southern Ethiopia, 2017**

| **Variables** | | **Anemia** | | **Adjusted OR (95% CI)** | | | | | | | | | | |
| --- | --- | --- | --- | --- | --- | --- | --- | --- | --- | --- | --- | --- | --- | --- |
|  |  |  |  | **Models** | | | | | | | | | | |
|  |  | Yes (%) | No (%) | I | II | P-value | III | P-value | IV | P-value | V | P-value | VI | P-value |
| **Individual child factors** | |  |  |  |  |  |  |  |  |  |  |  |  |  |
| Sex | Boys | 134 (29.4) | 322 (70.6) | - | 0.92 (0.66, 1.28) | 0.627 |  |  | - |  | - |  | 0.92 (0.66, 1.28) | 0.616 |
|  | Girls | 106 (29.9) | 248 (70.1) | - | 1.0 |  |  |  | - |  | - |  | 1.0 |  |
| Age in years | Mean (SD) | 11.4 (1.9) |  | - | 0.91 (0.81, 1.01) | 0.086 |  |  |  |  |  |  | 0.91 (0.81, 1.01) | 0.088 |
| Hand-washing with soap after use of latrine | Always | 21 (21.6) | 76 (78.4) | - | 1.0 |  |  |  | - |  | - |  | 1.0 |  |
|  | Sometimes or not always | 111 (23.8) | 356 (76.2) | - | 1.49 (0.81, 2.78) | 0.202 |  |  | - |  | - |  | 1.52 (0.82, 2.84) | 0.183 |
|  | Never | 108 (43.9) | 138 (56.1) | - | 2.15 (1.09, 4.25) | 0.027 |  |  | - |  | - |  | 2.09 (1.06, 4.14) | 0.033 |
| Taking a meal regularly before attending school | Yes | 219 (29.1) | 533 (70.9) |  | 0.65 (0.34, 1.24) | 0.188 |  |  | - |  | - |  | 0.64 (0.33, 1.23) | 0.178 |
|  | No | 21 (36.2) | 37 (63.8) |  | 1.0 |  |  |  | - |  | - |  | 1.0 |  |
| Stunting | No | 155 (27.9) | 400 (72.1) | - | 1.0 |  |  |  | - |  | - |  | 1.0 |  |
|  | Yes | 85 (33.3) | 170 (66.7) | - | 1.43 (0.99, 2.07) | 0.053 |  |  | - |  | - |  | 1.45 (1.005, 2.09) | 0.047 |
| *A. lumbricoides* | No | 174 (26.8) | 475 (73.2) | - | 1.0 |  |  |  | - |  | - |  | 1.0 |  |
|  | Yes | 64 (41.0) | 92 (59.0) | - | 1.74 (1.15, 2.62) | 0.009 |  |  | - |  | - |  | 1.73 (1.15, 2.62) | 0.009 |
| *T. trichiura* | No | 123 (26.5) | 342 (73.5) | - | 1.0 |  |  |  | - |  | - |  | 1.0 |  |
|  | Yes | 115 (33.8) | 225 (66.2) | - | 1.46 (1.04, 2.05) | 0.028 |  |  | - |  | - |  | 1.46 (1.04, 2.05) | 0.027 |
| *Hookworm* | No | 221 (28.8) | 547 (71.2) | - | 1.0 |  |  |  | - |  | - |  | 1.0 |  |
|  | yes | 17 (45.9) | 20 (54.1) | - | 1.78 (0.86, 3.70) | 0.120 |  |  | - |  | - |  | 1.76 (0.84, 3.65) | 0.131 |
| Received de-worming treatment in the past 6 months | Yes | 66 (37.9) | 108 (62.1) | - | 1.37(0.83, 2.26) | 0.219 |  |  | - |  | - |  | 1.40 (0.83, 2.37) | 0.204 |
|  | No | 174 (27.4) | 462 (72.6) | - | 1.0 |  |  |  | - |  | - |  | 1.0 |  |
| **Individual child factors** | |  |  |  |  |  |  |  |  |  |  |  |  |  |
| Mother’s education | No formal education | 216 (30.1) | 502 (69.9) | - | - |  | 1.40 (0.81, 2.43) | 0.234 | - |  | - |  | - |  |
|  | Primary and above | 24 (27.3) | 64 (72.7) | - | - |  | 1.0 |  | - |  | - |  | - |  |
| **Household factors** | |  |  |  |  |  |  |  |  |  |  |  |  |  |
| Wealth status | Poor | 73 (26.7) | 200 (73.3) | - | - |  | - |  | 0.85 (0.56, 1.28) | 0.437 | - |  | 0.82 (0.54, 1.26) | 0.369 |
|  | Middle | 92 (33.4) | 183 (66.6) | - | - |  | - |  | 1.04 (0.69, 1.58) | 0.837 | - |  | 1.12 (0.73, 1.72) | 0.606 |
|  | Rich | 75 (28.6) | 187 (71.4) | - | - |  | - |  | 1.0 |  | - |  | 1.0 |  |
| Family size | 1-4 | 21 (27.6) | 55 (72.4) | - | - |  | - |  | 1.0 |  | - |  | - |  |
|  | ≥5 | 219 (29.8) | 515 (70.2) | - | - |  | - |  | 1.11 (0.63, 1.95) | 0.711 | - |  | - |  |

CI: confidence interval; OR: odds ratio

**Table S8 Multivariate, multilevel, mixed-effect, regression analysis of anemia among schoolchildren in the Wonago district of southern Ethiopia, 2017 (Continued)**

| **Variables** | | **Anemia** | | **Adjusted OR (95% CI)** | | | | | | | | | | |
| --- | --- | --- | --- | --- | --- | --- | --- | --- | --- | --- | --- | --- | --- | --- |
|  |  |  |  | **Models** | | | | | | | | | | |
|  | | **Yes (%)** | **No (%)** | **I** | **II** | **P-value** | **III** | **P-value** | **IV** | **P-value** | **V** | **P-value** | **Model VI** | P-value |
| **Household factors** | |  |  |  |  |  |  |  |  |  |  |  |  |  |
| Using treated water at home | Yes | 32 (32.6) | 66 (67.4) | - | - |  | - |  | 0.98 (0.59, 1.61) | 0.938 | - |  | - |  |
|  | No | 208 (29.2) | 504 (70.8) | - | - |  | - |  | 1.0 |  | - |  | - |  |
| Food insecurity | No | 110 (27.4) | 291 (72.6) | - | - |  | - |  | 1.0 |  | - |  | 1.0 |  |
|  | Yes | 130 (31.8) | 279 (68.2) | - | - |  | - |  | 1.32 (0.87, 2.00) | 0.189 | - |  | 1.03 (0.65, 1.64) | 0.887 |
| Received food aid in the past 6 months | No | 227 (29.6) | 540 (70.4) | - | - |  | - |  | 1.0 |  | - |  | - |  |
|  | Yes | 13 (30.2) | 30 (69.8) | - | - |  | - |  | 0.73 (0.34, 1.54) | 0.404 | - |  | - |  |
| **School factors** | |  |  |  |  |  |  |  |  |  |  |  |  |  |
| Participates in school feeding program | No | 152 (38) | 248 (62.0) | - | - |  | - |  | - |  | 1.0 |  | 1.0 |  |
|  | Yes | 88 (21.5) | 322 (78.5) | - | - |  | - |  | - |  | 0.44 (0.18, 1.12) | 0.087 | 0.58 (0.23, 1.43) | 0.237 |
| **Variation and model fitness** | |  |  |  |  |  |  |  |  |  |  |  |  |  |
| Variance | School level |  |  | 0.32 | 0.20 |  | 0.34 |  | 0.32 |  | 0.16 |  | 0.13 |  |
|  | Class level |  |  | 0.20 | 0.21 |  | 0.19 |  | 0.21 |  | 0.20 |  | 0.19 |  |
| Intra-cluster correlation | School |  |  | 8.5 % | 5.4% |  | 8.8% |  | 8.4% |  | 4.4 |  | 3.6% |  |
|  | Class |  |  | 13.8 % | 11.0 % |  | 13.9 |  | 14.0% |  | 9.9% |  | 8.9% |  |
| **Model fitness** | |  |  |  |  |  |  |  |  |  |  |  |  |  |
| -2 Log likelihood | |  |  | 929 | 888 |  | 926 |  | 926 |  | 927 |  | 880 |  |
| Akaike information criterion | |  |  | 936 | 914 |  | 934 |  | 944 |  | 935 |  | 914 |  |
| Area under the curve = 0.75 | |  |  |  |  |  |  |  |  |  |  |  |  |  |

CI: confidence interval; OR: odds ratio
