# Supplementary material for: Concurrent anemia and stunting among schoolchildren in Wonago district in southern Ethiopia: a cross-sectional multilevel analysis
Source: PeerJ. 2021 May 6;9:e11158. doi: 10.7717/peerj.11158 (PMC8106909; doi:10.7717/peerj.11158)
Supplement: Supplemental Information 10 [file peerj-09-11158-s010.docx]

**Table S9 Multivariate, multilevel, mixed-effect, regression analysis of stunting among schoolchildren in the Wonago district of southern Ethiopia, 2017**

| **Variables** | | **Stunting** | | **Adjusted OR (95% CI)** | | | | | | | | | | |
| --- | --- | --- | --- | --- | --- | --- | --- | --- | --- | --- | --- | --- | --- | --- |
|  |  |  |  | **Models** | | | | | | | | | | |
| **Individual child factors** | | **Yes (%)** | **No (%)** | **I** | **II** | **P-value** | **III** | **P-value** | **IV** | **P-value** | **V** | **P-value** | **VI** | **P-value** |
| Sex | Boys | 167 (34.6) | 316 (65.4) | - | 1.30 (0.94, 1.81) | 0.109 |  |  | - |  | - |  | 1.29 (0.93, 1.79) | 0.134 |
|  | Girls | 111 (29.4) | 267 (70.6) | - | 1.0 |  | - |  | - |  | - |  | 1.0 |  |
| Age in years | Mean (SD) | 11.4 (1.9) |  | - | 1.47 (1.25, 1.73) | 0.000 | - |  | - |  | - |  | 1.47 (1.24, 1.74) | 0.000 |
| Trim nail every week | Yes | 200 (30.1) | 465 (69.9) | - | 0.78 (0.50, 1.21) | 0.261 | - |  | - |  | - |  | - |  |
|  | No | 78 (39.8) | 118 (60.2) | - | 1.0 |  | - |  | - |  | - |  | - |  |
| Hand-washing with soap after latrine | Always | 22 (21.4) | 81 (78.6) | - | 1.0 |  | - |  | - |  | - |  | 1.0 |  |
|  | Sometimes or not always | 167 (34.3) | 320 (65.7) | - | 1.98 (1.12, 3.52) | 0.019 | - |  | - |  | - |  | 2.09 (1.17, 3.71) | 0.012 |
|  | Never | 89 (32.8) | 182 (67.2) | - | 1.67 (0.88, 3.17) | 0.114 | - |  | - |  | - |  | 1.73 (0.88, 3.41) | 0.111 |
| Anaemia | No | 170 (29.8) | 400 (70.2) | - | 1.0 |  | - |  | - |  | - |  | 1.0 |  |
|  | Yes | 85 (35.4) | 155 (64.6) | - | 1.47 (1.03, 2.11) | 0.033 | - |  | - |  | - |  | 1.45 (1.01, 2.09) | 0.044 |
| Head lice | Yes | 123 (36.0) | 219 (64.0) | - | 1.40 (1.01, 1.95) | 0.043 | - |  | - |  | - |  | 1.41 (1.003, 1.97) | 0.048 |
|  | No | 155 (29.9) | 364 (70.1) | - | 1.0 |  | - |  | - |  | - |  | 1.0 |  |
| **Individual child factors** | |  |  |  |  |  |  |  |  |  |  |  |  |  |
| Mother’s education | No formal education | 250 (32.8) | 511 (67.2) | - | - |  | 1.35 (0.82, 2.22) | 0.243 | - |  | - |  | - |  |
|  | Primary and above | 27 (28.1) | 69 (71.9) | - | - |  | 1.0 |  | - |  | - |  | - |  |
| **Household factors** | |  |  |  |  |  |  |  |  |  |  |  |  |  |
| Wealth | Poor | 104 (36.2) | 183 (63.8) | - | - |  | - |  | 1.38 (0.96, 1.98) | 0.078 | - |  | 1.33 (0.89, 1.98) | 0.156 |
|  | Middle | 88 (29.6) | 209 (70.4) | - | - |  | - |  | 0.97 (0.67, 1.39) | 0.863 | - |  | 0.99 (0.66, 1.51) | 0.991 |
|  | Rich | 86 (31.1) | 191 (68.9) | - | - |  | - |  | 1.0 |  | - |  | 1.0 |  |
| Family size | 1-4 | 19 (24.4) | 59 (75.6) | - | - |  | - |  | 1.0 |  | - |  | 1.0 |  |
|  | ≥5 | 259 (33.1) | 524 (66.9) | - | - |  | - |  | 1.54 (0.89, 2.65) | 0.120 | - |  | 1.58 (0.87, 2.87) | 0.130 |
| Using treated water at home | Yes | 22 (20.2) | 87 (79.8) | - | - |  | - |  | 0.49 (0.30, 0.82) | 0.006 | - |  | 0.52 (0.30, 0.90) | 0.020 |
|  | No | 256 (34.0) | 496 (66.0) | - | - |  | - |  | 1.0 |  | - |  | 1.0 |  |
| Food insecurity | No | 149 (35.1) | 275 (64.9) | - | - |  | - |  | 1.0 |  | - |  | 1.0 |  |
|  | Yes | 129 (29.5) | 308 (70.5) | - | - |  | - |  | 0.76 (0.57, 1.03) | 0.074 | - |  | 0.69 (0.46, 1.04) | 0.080 |
| Received food aid in the past 6 months | No | 270 (33.2) | 544 (66.8) | - | - |  | - |  | 1.0 |  | - |  | 1.0 |  |
|  | Yes | 8 (17.0) | 39 (83.0) | - | - |  | - |  | 0.42 (0.19, 0.93) | 0.032 | - |  | 0.38 (0.15, 0.99) | 0.049 |
| **School factors** | |  |  |  |  |  |  |  |  |  |  |  |  |  |
| Participates in school feeding program | No | 139 (32.3) | 292 (67.7) | - | - |  |  |  | - |  | 1.0 |  | 1.0 |  |
|  | Yes | 139 (32.3) | 291 (67.7) | - | - |  | - |  | - |  | 1.003 (0.74, 1.35) | 0.984 | 0.71 (0.40, 1.25) | 0.237 |
| **Variation and model fitness** | |  |  |  |  |  |  |  |  |  |  |  |  |  |
| Variance | School level |  |  | NS | NS |  | 0.01 |  | NS |  | NS |  | NS |  |
|  | Class level |  |  | 0.013 | 0.22 |  | 0.02 |  | NS |  | 0.013 |  | 0.18 |  |
| Intra-cluster correlation | School |  |  | NS | NS |  | 0.4% |  | NS |  | NS |  | NS |  |
|  | Class |  |  | 0.4 % | 6.2% |  | 0.8% |  | NS |  | 0.4% |  | 5.2% |  |
| **Model fitness** | |  |  |  |  |  |  |  |  |  |  |  |  |  |
| -2 Log likelihood | |  |  | 1082 | 944 |  | 1076 |  | 1058 |  | 1082 |  | 926 |  |
| Akaike information criterion | |  |  | 1089 | 961 |  | 1085 |  | 1073 |  | 1089 |  | 956 |  |
| Area under the curve =0.72 | |  |  |  |  |  |  |  |  |  |  |  |  |  |

CI: confidence interval; NS: Not significant; OR: odds ratio
