# Supplementary material for: Concurrent anemia and stunting among schoolchildren in Wonago district in southern Ethiopia: a cross-sectional multilevel analysis
Source: PeerJ. 2021 May 6;9:e11158. doi: 10.7717/peerj.11158 (PMC8106909; doi:10.7717/peerj.11158)
Supplement: Supplemental Information 11 [file peerj-09-11158-s011.docx]

**Table S10 Multivariate, multilevel, mixed-effect, regression analysis of anemia, stunting, and CAS among schoolchildren in the Wonago district of southern Ethiopia, 2017**

| **Variables** | | **Adjusted OR (95% CI)** | | | | | |
| --- | --- | --- | --- | --- | --- | --- | --- |
| **Individual child factors** | | **Anemia** | **P-value** | **Stunting** | **P-value** | **CAS** | **P-value** |
| Sex | Boys | 0.92 (0.66, 1.28) | 0.62 | 1.29 (0.93, 1.79) | 0.13 | 0.84 (0.51, 1.39) | 0.499 |
|  | Girls | 1.0 |  | 1.0 |  | 1.0 |  |
| Age |  | 0.91 (0.81, 1.01) | 0.09 | 1.47 (1.24, 1.74) | 0.000 | 1.39 (1.13, 1.71) | 0.002 |
| Hand washing with soap after latrine | Always | 1.0 |  | 1.0 |  | 1.0 |  |
|  | Sometimes or not always | 1.52 (0.82, 2.84) | 0.18 | 2.09 (1.17, 3.71) | 0.01 | 4.30 (1.21, 15.3) | 0.02 |
|  | Never | 2.09 (1.06, 4.14) | 0.03 | 1.73 (0.88, 3.41) | 0.11 | 3.10 (0.82, 11.8) | 0.09 |
| Walking bare foot | Always | - |  | - |  | 10.4 (2.77, 39.1) | 0.001 |
|  | Sometimes | - |  | - |  | 1.18 (0.68, 2.05) | 0.55 |
|  | Never | - |  | - |  | 1.0 |  |
| Head lice | Yes | - |  | 1.41 (1.003, 1.97) | 0.04 | 1.71 (1.01, 2.92) | 0.04 |
|  | No | - |  | 1.0 |  | 1.0 |  |
| *A. lumbricoides* | No | 1.0 |  | - |  | - |  |
|  | yes | 1.73 (1.15, 2.62) | 0.009 | - |  | - |  |
| *T. trichiura* | No | 1.0 |  | - |  | 1.0 |  |
|  | Yes | 1.46 (1.04, 2.05) | 0.02 | - |  | 1.74 (1.05, 2.88) | 0.03 |
| **Household factors** | |  |  |  |  |  |  |
| Using treated drinking water | Yes | - |  | 0.52 (0.30, 0.90) | 0.02 | 0.32 (0.11, 0.97) | 0.04 |
|  | No | - |  | 1.0 |  | 1.0 |  |
| **School factors** | |  |  |  |  |  |  |
| Participates in school meal programme | No | 1.0 |  | 1.0 |  | 1.0 |  |
|  | Yes | 0.58 (0.23, 1.43) | 0.23 | 0.71 (0.40, 1.25) | 0.23 | 0.29 (0.07, 1.17) | 0.08 |
| **Variation and model fitness** |  |  |  |  |  |  |  |
| Intra-cluster correlation | School | 3.6% |  | NS |  | 6.8% |  |
|  | Class | 8.9% |  | 5.2 |  | 19% |  |
| -2 Log likelihood |  | 880 |  | 926 |  | 466 |  |
| AIC |  | 914 |  | 956 |  | 502 |  |
| AUC |  | 0.75 |  | 0.72 |  | 0.81 |  |

AIC: Akaike information criterion; AUC: area under the curve; CAS: concurrent anemia and stunting; CI: confidence interval; OR: odds ratio. Model VI for anemia included individual (sex, age, hand washing with soap after latrine use, meal habit before school, stunting, *A. lumbricoides, T. trichiura*, hookworm, de-worming treatment), household (wealth status, food insecurity), and school (participation in school meal programme) factors. Model VI for stunting included individual (sex, age, hand washing with soap after latrine use, anemia, head lice), household (wealth status, family size, using treated water at home, food insecurity, access to food aid in the past 6 months), and school (participation in school meal programme) factors. Model VI for CAS included individual child (sex, age, hand washing with soap after latrine use, walking barefoot, *T. trichiura*, and head lice), parent (mother’s education), household (wealth status, family size, using treated water at home, access to food aid in the past 6 months), and school (participation in school meal programme) factors.
