# Supplementary material for: Concurrent anemia and stunting among schoolchildren in Wonago district in southern Ethiopia: a cross-sectional multilevel analysis
Source: PeerJ. 2021 May 6;9:e11158. doi: 10.7717/peerj.11158 (PMC8106909; doi:10.7717/peerj.11158)
Supplement: Supplemental Information 4 [file peerj-09-11158-s004.docx]

**Table S3 Affirmative responses to items on the Household Food Insecurity Access Scale (HFIAS) in the Wonago district of southern Ethiopia, 2017 (n=861)**

| **HFIAS questions** | | **Frequency** | **Percent** |
| --- | --- | --- | --- |
| Q1. | Worry for food | 437 | 50.8 |
| Q2. | Unable to eat preferred foods | 358 | 41.6 |
| Q3. | Eat a limited variety of foods | 291 | 33.8 |
| Q4. | Eat foods that you did not want to eat | 193 | 22.4 |
| Q5. | Eat a smaller meal | 121 | 14.1 |
| Q6. | Eat fewer meals in a day | 48 | 5.6 |
| Q7. | No food to eat of any kind | 29 | 3.4 |
| Q8. | Go to sleep at night hungry | 17 | 2.0 |
| Q9. | Go day and night without eating anything | 10 | 1.2 |
| Prevalence of food insecurity | Food secure | 424 | 49.2 |
|  | Mild food insecurity | 252 | 29.3 |
|  | Moderate food insecurity | 155 | 18.0 |
|  | Severe food insecurity | 30 | 3.5 |

Dear professor,

Thank you so much for your comments and suggestions. I will send the revised text. If you can, would you please look at the introduction because I also added some text.

One question: As you observed, editors asked for diagram showing interrelationship between variables and I used to show interrelationship in bar graph. Is it good graph?

Another question: in the discussion section lines: 390-391: CAS was found to be a moderate public health problem (De Benoist et al., 2008; de Onis et al., 2019) among schoolchildren in southern Ethiopia. There is no cut value to classify as low, moderate or high public health problem for co-existence of anemia and stunting. However, I found two reference for these classification for anemia or stunting.

*What criteria justifies a qualification of "moderate", "high" or else and what are the references?*
